# Interpreting the Outcomes of Automated Internet-Based Randomized Trials: Example of an International Smoking Cessation Study

**DOI:** 10.2196/jmir.1829

**Published:** 2012-02-07

**Authors:** Yan Leykin, Adrian Aguilera, Leandro D Torres, Eliseo J Pérez-Stable, Ricardo F Muñoz

**Affiliations:** ^1^Department of PsychiatryUniversity of California, San FranciscoSan Francisco, CAUnited States; ^2^School of Social WelfareUniversity of California, BerkeleyBerkeley, CAUnited States; ^3^San Francisco General HospitalUniversity of California, San FranciscoSan Francisco, CAUnited States; ^4^Division of General Internal MedicineDepartment of MedicineUniversity of California, San FranciscoSan Francisco, CAUnited States; ^5^Helen Diller Family Comprehensive Cancer CenterUniversity of California, San FranciscoSan Francisco, CAUnited States

**Keywords:** Smoking cessation, tobacco use, Internet intervention, evidence-based intervention, attrition, effectiveness trial

## Abstract

**Background:**

Smoking is one of the largest contributors to the global burden of disease. Internet interventions have been shown to reduce smoking rates successfully. However, improved methods of evaluating effectiveness need to be developed for large-scale Internet intervention trials.

**Objective:**

To illustrate a method to interpret outcomes of large-scale, fully automated, worldwide Internet intervention trials.

**Methods:**

A fully automated, international, Internet-based smoking cessation randomized controlled trial was conducted in Spanish and English, with 16,430 smokers from 165 countries. The randomized controlled trial replicated a published efficacy trial in which, to reduce follow-up attrition, 1000 smokers were followed up by phone if they did not provide online follow-up data.

**Results:**

The 7-day self-reported abstinence rates ranged from 36.18% (2239/6189) at 1 month to 41.34% (1361/3292) at 12 months based on observed data. Given high rates of attrition in this fully automated trial, when participants unreachable at follow-up were presumed to be smoking, the abstinence rates ranged from 13.63% (2239/16.430) at 1 month to 8.28% (1361/16,430) at 12 months. We address the problem of interpreting results with high follow-up attrition rates and propose a solution based on a smaller study with intensive phone follow-up.

**Conclusions:**

Internet-based smoking cessation interventions can help large numbers of smokers quit. Large-scale international outcome studies can be successfully implemented using automated Internet sites. Interpretation of the studies’ results can be aided by extrapolating from results obtained from subsamples that are followed up by phone or similar cohort maintenance methods.

**Trial Registration:**

ClinicalTrials.gov NCT00721786; http://clinicaltrials.gov/ct2/show/NCT00721786 (Archived by WebCite at http://www.webcitation.org/63mhoXYPw)

## Introduction

One billion tobacco-related deaths are projected for the 21st century, 80% of which will occur in low- and middle-income countries [1]. Current health care approaches for smoking cessation include nicotine replacement therapy (eg, nicotine patch or gum; quit rate: 14%–24%), other prescription medication (eg, bupropion or varenicline; quit rates: up to 25% and 30%, respectively), and psychosocial interventions (quit rates: 15%–27%) [2-4]. However, these are expensive, unavailable to many who need them, and consumable in terms of both the actual product and the time of clinicians providing treatment. Highly scalable, widely accessible, nonconsumable, evidence-based smoking cessation methods must be developed and evaluated to reduce smoking rates. Large-scale automated self-help Internet interventions are one such method; however, the implementation of these trials presents several practical and methodological challenges. This report presents a promising approach to the implementation of automated, worldwide Internet randomized trials and the interpretation of their results, with attention to assessing effectiveness in addition to efficacy.

### Limitations of Current Smoking Cessation Approaches

Internet-based interventions have several advantages, including time and cost effectiveness, almost unlimited scalability, increased intervention fidelity, ease of updates and expansions to conform to the most up-to-date research, and the ability to make them available across the world. With such penetration, even small improvements in likelihoods of quitting smoking can have profound effects on public health relative to the cost of the intervention (see the RE-AIM framework [5-11]). Reports indicate that, in fact, Internet interventions can obtain quit rates comparable with those of other treatment modalities [12-19].

A key benefit of Internet interventions is the automation of delivery. However, with few exceptions [13,18], most trials rely on live personnel to conduct follow-up assessments via phone or email contacts with participants. Insofar as interaction with live personnel may affect outcomes, these trials depart from the fully automated framework. The two main reasons for sacrificing the benefits of automation and fidelity of administration, while incurring considerable costs to conduct live follow-up, are attrition and the analytical convention of imputing smoking status to missing data when presenting cessation trial outcomes.

The progression of Internet intervention studies to address a particular health problem at a worldwide level generally begins with face-to-face clinical trials. The interventions developed at this stage are then adapted for delivery via the Web. Online randomized controlled trials with strong cohort maintenance efforts, such as using staff to send personalized email or to make phone calls to reach participants who do not respond to automated follow-up assessments, can provide estimates of outcome that approximate traditional methods. We suggest that the next step ought to be very-large-scale randomized trials, conducted in a fully automated fashion, to reflect as closely as possible the routine dissemination of Internet interventions that can be made available to anyone in the world, with minimal staffing. However, such large-scale trials generally cannot afford individual live follow-up. This report presents a method that may help researchers in the field to estimate effectiveness data of self-help automated interventions.

### Attrition

The motivation needed to enter traditional face-to-face trials is high: people either actively seek them out, respond to an advertisement by calling and visiting a clinic, or are directly recruited from preexisting registries based on demographic, behavioral, or clinical factors. In contrast, those signing up for a Web-based trial generally do so via a Web search and clicking on a link. Of the thousands who visit the website, few will elect to join, fewer still will make adequate use of the intervention, and only a minority will respond to automated follow-up invitations. The difference in effort involved to enter an Internet trial versus a face-to-face trial makes comparisons between the two problematic. Website visitors are more akin to persons reading an advertisement for a trial, most of whom will not actually call or visit the study clinic. Those filling out an online eligibility questionnaire are similar to those calling a phone number to inquire more about a traditional outcome study. Signing up for an online trial takes little effort; although many online participants are likely curious about the Internet trial, they may not be as committed to participating as those signing up after traveling to a study clinic. Once people enter into the study, it is extremely easy for them to drop out of an Internet trial, since there has been no direct personal contact with study staff. Researchers in the field need to reconsider how best to interpret findings that involve large attrition to systematically study the effectiveness of Internet interventions as they would be routinely used in practice, rather than as part of a well-staffed randomized controlled trial.

Attrition is a recognized concern in Internet trials [20], which affects interpretation of results in two ways: (1) if most participants drop out, the remaining sample is highly self-selected and may not be representative of the original visitors, and (2) if participants do not complete the intervention, but respond to follow-up, the outcome data won’t represent the intervention’s potential. Of course, there are also parallels in face-to-face trials: participants are also highly self-selected and not a representative sample of all who have the disorder being treated.

### The “Missing = Smoking” Convention

The usual strategy for determining quit rates in a cessation trial is the *missing = smoking* (M=S) convention, a variant of the intent-to-treat analysis, which presumes that all participants unreachable at follow-up are smoking. This is similar to the last observation carried forward (LOCF) convention; however, the M=S convention is more conservative, for two reasons. First, the LOCF convention permits *responded-to-treatment* observations to be carried forward as well as *nonresponse*, whereas M=S presumes that every dropout is a treatment failure. Second, because cessation trial outcomes are dichotomous (quit vs did not quit), the degree of response/nonresponse (eg, fewer cigarettes) cannot be captured by M=S.

 The outcomes of cessation trials therefore largely depend on the completeness of follow-up data. For example, suppose the *true* quit rate for a hypothetical intervention is 20%. Three trials assessing the effect of that intervention with follow-up rates of 100%, 70%, and 40% would yield M=S outcomes of 20%, 14%, and 8%, respectively, prompting widely differing conclusions about efficacy. Since 14%–22% can be expected with a nicotine patch [4] and 4%–8% can be obtained with a placebo patch, the possible M=S outcome implications vary significantly. Because automated Internet trials have inherently high dropout rates, the M=S convention may be more reflective of follow-up success than of treatment efficacy.

### An Illustration of an Interpretable Internet-Based Cessation Trial

With live follow-up (eg, phone calls), it is possible for geographically limited Internet trials to obtain follow-up rates of up to 78%, which is comparable with face-to-face trials [21]. However, live follow-ups for large-scale, worldwide Internet trials are costly and logistically difficult. Conversely, allowing the logistical limitations of live follow-up procedures to constrain the number of participants compromises scope and reach, limiting the public health applications of an Internet trial.

We propose one possible model of structuring an Internet trial that may help assess effectiveness of a trial once efficacy is established. In 2009, Muñoz and colleagues reported on the outcome of a Web-based smoking cessation trial conducted in Spanish and English (n = 1000) [14]. Live follow-up was used with those who did not provide data after an automated reminder, obtaining follow-up rates of 68% at 12-month follow-up. At 12 months, 20% of Spanish- and 21% of English-speaking participants were no longer smoking (M=S). After random allocation of the first 1000 participants, live follow-up ended, but the rest of the online intervention study was left exactly the same, with the goal of conducting a larger trial to demonstrate the demand for and the reach of the intervention as delivered in a fully automated format. Here, we report on the results of the fully automated portion of that trial.

Conducting a smaller and logistically feasible live follow-up trial followed by or concurrently with a larger fully automated trial can address the concerns of cost versus scope mentioned previously. The goal of the current study was therefore to illustrate the use of this approach in interpreting the outcomes of a fully automated trial. We used the outcomes of the Muñoz et al [14] trial to interpret the results of the current fully automated trial, and we tested three hypotheses to determine whether a more complex intervention would increase quit rates.

## Methods

### Participants

Recruitment procedures were the same as described elsewhere [14]. Google AdWords ads were the main means for recruiting participants. Eligible participants were 18+ years of age, smoking 5+ cigarettes per day, with regular (1+ times/week) access to email and Internet, intending to quit in the next month. Of the visitors screened for eligibility, 16,475/78,623 (20.95%) were ineligible, 1052/78,623 (1.34%) were <18 years old, 2738/78,623 (3.48%) smoked <5 cigarettes/day, 9875/78,623 (12.56%) were not ready to quit, and 4646/78,623 (5.91%) had no email address. Participants were not paid for their participation in the study. Participants were recruited from November 2005 to September 2009.

### Study Procedures

Study procedures are described in detail elsewhere [14]. Briefly, visitors to the site completed brief demographics and eligibility questionnaires. Eligible participants viewed and e-signed a consent document, which detailed the study procedures, including randomization. Consenting participants completed baseline questionnaires. To select out one-time visitors, participants were asked to return 3 times over the next 7 days and report the number of cigarettes smoked. Those meeting this requirement set their quit dates, were automatically randomly assigned to 1 of 4 conditions, and were given access to the website. Participants were sent automated follow-up assessment emails at 1, 3, 6, and 12 months after their quit date. Only self-reported smoking data were gathered, for three reasons: (1) biochemical verification was not feasible for a very large worldwide trial, (2) the fully automated nature of the trial precluded additional participant contact, and (3) there is growing evidence that self-report is sufficient for nonintensive interventions [22-24].

The only difference from the procedures described in the 2009 [14] paper, wherein research assistants either called or emailed those who did not submit follow-up data after the automated email contact, was that in the present study we did not use live follow-up.

### Study Conditions

As in the 2009 trial [14], participants were randomly assigned to 1 of 4 conditions. The website used a preprogrammed algorithm for random assignment using stratiﬁed randomization, with gender and history of major depressive episodes (MDEs) as stratification variables. Condition 1 contained the most basic elements, and conditions 2–4 incrementally added further elements.

The 4 arms (conditions) of the trial were the following:

1. A noninteractive, static smoking cessation guide (Guía para dejar de fumar [25-27]), a cigarette counter, and an online journal.

2. Condition 1, plus individually timed email messages: preprogrammed emails with links to sections of smoking cessation guide timed to quit date [28].

3. Condition 2, plus an 8-session cognitive–behavioral mood management course (based on Lewinsohn et al [29]).

4. Condition 3, plus a virtual participant-driven, unmoderated support group (an asynchronous bulletin board).

### Hypotheses

We retained three specific hypotheses regarding the outcome of the intervention from the 2009 [14] study and tested them in the fully automated sample: (1) conditions 2, 3, and 4 will outperform condition 1, (2) condition 4 will obtain the best quit rates, followed by condition 3, followed by condition 2, followed by condition 1, and (3) conditions 3 and 4 (containing mood management) will outperform conditions 1 and 2.

### Measures

A *d* *emographic questionnaire* included age, gender, race/ethnicity, education, income, and marital status.

A *s* *moking questionnaire* included age when the participant started smoking, age when smoking regularly, number of cigarettes per day, confidence in quitting, and smoking exposure.

The *Fagerström Test for Nicotine Dependence* (FTND) [30] is a commonly used 6-item test of nicotine dependence, with a range from 0 to 10.

The *MDE Screener* (Mood Screener) [31] screens for the presence of the 9 symptoms of current and past MDEs according to the *Diagnostic and Statistical Manual of Mental Disorders*, 4th edition, as well as for criterion C (significant impairment in functioning). This instrument has been shown to have good agreement with the PRIME-MD and with clinician-administered interviews [32-34].

The *Center for Epidemiologic Studies Depression* scale (CES-D) [35] is a 20-item self-report scale designed to measure the current level of depressive symptoms.

### Statistical Analyses

To test hypotheses 1 (condition 1 will result in worse outcomes) and 3 (conditions with mood management will result in better outcomes), we conducted repeated binary logistic regressions. The quit rates were predicted from the intervention condition assignment (1 versus others for hypothesis 1; 1 and 2 versus 3 and 4 for hypothesis 3), covarying participant demographic characteristics (gender, age, education, and race), language (English or Spanish), depression (CES-D score and presence of current or past MDE), and level of addiction (FTND). We conducted these analyses twice: once with the M=S assumption, and the other with observed data (without the M=S assumption).

To test hypothesis 2—that intervention conditions would yield incrementally better outcomes—we constructed binary logistic regression models, predicting the 7-day quit rate at 1, 3, 6, and 12 months. The model predictors were the same as those used for repeated measures analyses, described above. As above, these analyses were conducted twice: once with the M=S assumption, and the other with observed data (without the M=S assumption).

 Due to the considerable size of the sample (n = 16,430), we elected to report significance only if we obtained a *P* value less than .01, to reduce type I error.

## Results

### Sample Characteristics

Participants were 16,430 smokers (3332 English- and 13,098 Spanish-speaking), aged 18 to 84 (mean 36.2, SD 10.7), from 165 countries. The three most-represented countries for English speakers were the United States (n = 1251), India (n = 358), and South Africa (n = 306). The three most-represented Spanish-speaking countries were Spain (n = 4341), Argentina (n = 2513), and Mexico (n = 2100). Just over half of participants were men (8638/16,349, 52.84%), and most were well educated (12,628/16,379, 77.10% with at least some college education), gainfully employed (12,960/16,415, 78.95% at least part-time), and married or living as married (8846/16,403, 53.93%).

Participants reported having smoked for 20.6 years, on average (SD 10.9), smoking on average 1 pack per day (mean 19.6, SD 9.9 cigarettes). The average age at first cigarette was 15.6 (SD 3.2) years, and the average age of smoking regularly (first 5 packs) was 18.6 (SD 4.3). The average level of nicotine dependence, as measured by the FTND, was 5.2 (SD 2.5), indicating moderate dependence, and similar to face-to-face smoking cessation trials [36-39].

Participant characteristics for each condition are shown in Table 1.

**Table 1:** Participant characteristics, by condition^a^

	Condition 1 (n = 4118)	Condition 2 (n = 4097)	Condition 3 (n = 4110)	Condition 4 (n = 4105)	*P* value^b^
Male, n (%)	2168/4102 (52.85%)	2150/4080 (52.70%)	2165/4088 (52.96%)	2155/4079 (52.83%)	1.00
Age (years), mean (SD)	36.1 (11.4)	36.3 (11.8)	36.5 (14.5)	36.4 (13.6)	.47
Some college or more, n (%)	3167/4107 (77.11%)	3141/4088 (76.83%)	3173/4091 (77.56%)	3147/4093 (76.89%)	.93
White, n (%)	2803/4086 (68.60%)	2801/4069 (68.84%)	2796/4076 (68.60%)	2802/4075 (68.76%)	.37
Spanish-speaking, n(%)	3284/4118 (79.75%)	3263/4097 (79.64%)	3275/4110 (79.68%)	3276/4105 (79.81%)	1.00
Employed, n (%)	3217/4115 (78.18%)	3237/4091 (79.12%)	3268/4109 (79.53%)	3238/4100 (78.98%)	.36
Married or partnered, n (%)	2206/4112 (53.65%)	2209/4089 (54.02%)	2234/4103 (54.45%)	2197/4099 (53.60%)	.86
CES-D^c^ score, mean (SD)	16.9 (12.1)	17.0 (12.0)	16.8 (12.4)	16.9 (12.4)	.74
Current or past major depressive episode, n (%)	1276/4109 (31.05%)	1275/4091 (31.17%)	1280/4103 (31.20%)	1277/4101 (31.14%)	1.00
Cigarettes/day, mean (SD)	19.4 (9.9)	19.8 (10.2)	19.5 (10.1)	19.6 (9.7)	.36
Age started smoking (years), mean (SD)	15.5 (3.2)	15.6 (3.2)	15.5 (3.2)	15.6 (3.4)	.46
Age regular smoker (years), mean (SD)	18.6 (4.4)	18.5 (4.0)	18.6 (4.3)	18.7 (4.4)	.19
Years smoked, mean (SD)	20.5 (10.8)	20.6 (10.9)	20.7 (10.9)	20.6 (11.0)	.81
FTND^d^ score, mean (SD)	5.2 (2.5)	5.3 (2.5)	5.2 (2.5)	5.2 (2.5)	.41

^a^ Conditions were as follows: condition 1: a noninteractive smoking cessation guide, cigarette counter, and an online journal; condition 2: condition 1, plus individually timed email messages; condition 3: condition 2, plus an 8-session cognitive–behavioral mood management course; and condition 4: condition 3, plus a virtual participant-driven support group.

^b^ *P* values were determined via 1-way analyses of variance for continuous variables, and via Pearson chi-squares for categorical variables.

^c^ Center for Epidemiologic Studies Depression scale.

^d^ Fagerström Test for Nicotine Dependence.

### Attrition

**Figure 1:**
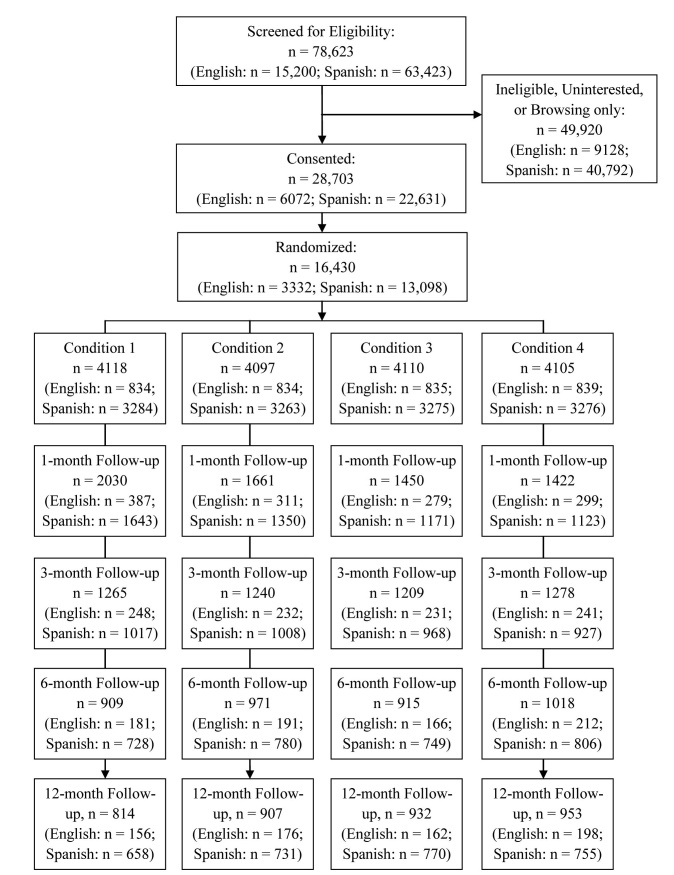
CONSORT diagram for progression of participants through the fully automated Internet stop smoking trial.

The progression of participants through the study is outlined in Figure 1. Of the over 150,000 participants who visited our website, 78,623 provided enough data to evaluate their eligibility, 28,703 signed consent, and 16,430 completed baseline assessments and the washout period and underwent random assignment.

The current study relied solely on automated emailed reminders to obtain follow-up data. For month 1 follow-up, 6563/16,430 (40.0%) participants provided data. This number was reduced to 4992/16,430 (30.38%), 3813/16,430 (23.21%), and 3606/16,430 (21.95%) for follow-ups at months 3, 6, and 12, respectively. These numbers were comparable with those obtained in the earlier [14] study, where 38%, 30%, 27%, and 23% of participants who never received any live follow-up returned at months 1, 3, 6, and 12, respectively.

### Abstinence Rates

Based on observed data, 1 month after enrollment, 36.18% (2239/6189) reported not having smoked in the past 7 days (Table 2). A similar proportion of participants reported 7-day abstinence at months 3 (1797/4566, 39.36%), 6 (1478/3508, 42.13%), and 12 (1361/3292, 41.34%). A somewhat smaller proportion of participants reported not having smoked in the past 30 days (1640/6182, 26.53%; 1465/4562, 32.11%; 1243/3504, 35.47%; and 1211/3286, 36.85% at 1, 3, 6, and 12 months, respectively). Using the M=S convention, the cessation rates observed in this study were modest. The 7-day abstinence rates at 1, 3, 6, and 12 months, respectively, were 13.63% (2239/16,430), 10.94% (1797/16,430), 9.00% (1478/16,430), and 8.28% (1361/16,430). The respective 30-day abstinence rates were 9.98% (1640/16,430), 8.92% (1465/16,430), 7.57% (1243/16,430), and 7.37% (1211/16,430). In the 45 months of this intervention being available online, 3,489 individuals reported having quit for at least 7 days (about 18 per week over the course of the study), and 2,786 individuals (about 14 per week) reported having quit for at least 30 days.

**Table 2:** Overall self-reported abstinence rates (% quit) in an online sample of 16,430 consented smokers

		1-month follow-up		3-month follow-up		6-month follow-up		12-month follow-up	
Completed follow-ups, n (%)		6563/16,430 (39.95%)		4992/16,430 (30.38%)		3813/16,430 (23.21%)		3606/16,430 (21.95%)	
		7 days	30 days	7 days	30 days	7 days	30 days	7 days	30 days
**Observed **								
	n	2239/6189	1640/6182	1797/4566	1465/4562	1478/3508	1243/3504	1361/3292	1211/3286
	%	36.18%	26.53%	39.36%	32.11%	42.13%	35.47%	41.34%	36.85%
**M=S^a^**								
	n	2239/16,430	1640/16,430	1797/16,430	1465/16,430	1478/16,430	1243/16,430	1361/16,430	1211/16,430
	%	13.63%	9.98%	10.94%	8.92%	9.00%	7.57%	8.28%	7.37%

^a^ Missing observations are presumed to be smoking.

### Intervention Conditions

We noted several differences between treatment conditions (Table 3). With observed data (ie, without the M=S assumption), at 1-month follow-up, condition 1 performed significantly poorer than all other conditions, in partial support for hypothesis 1 (Wald χ^2^_3_ = 80.7, *P* < .001). No significant differences between conditions were noted at months 3 (Wald χ^2^_3_ = 10.3, *P* = .02), 6 (Wald χ^2^_3_ = 5.8, *P* = .12), and 12 (Wald χ^2^_3_ = 7.5, *P* = .06). With M=S analyses, significant differences were observed at months 6 (Wald χ^2^_3_ = 14.8, *P* = .002) and 12 (Wald χ^2^_3_ = 13.0, *P* = .005), such that conditions 2 and 4 outperformed conditions 1 and 3.

Observing the quit rates, it is clear that hypothesis 2—that conditions would result in incremental improvements in quit rates—is not supported. To test the two other hypotheses, we conducted repeated-measures logistic regressions, with the same covariates as in the simple logistic regressions above. Hypothesis 1 was largely supported. With observed data, condition 1 resulted in lower quit rates than conditions 2, 3, and 4 (Wald χ^2^_1_ = 30.1, *P* < .001, beta = –.28, 95% confidence interval [CI], –.39 to –.18). A similar result was observed with M=S data, though the result did not cross the significance level set for this study (Wald χ^2^_1_ = 6.1, *P* = .01, beta *=* –.12, 95% CI –.21 to –.02). Hypothesis 3—that mood management conditions would result in higher quit rates—was supported only with observed data (Wald χ^2^_1_ = 9.5, *P* = .002, beta = –.14, 95% CI –.23 to –.05), but not with the M=S data (Wald χ^2^_1_ = .0, *P* = .96, beta = .00, 95% CI –.08 to .78).

**Table 3:** 7-day quit rates (n, %) by intervention condition

		Condition^a^				*P* value
		1 (cessation guide)	2 (1 + email messages)	3 (2 + mood management)	4 (3 + virtual group)	
**Observed**						
	Month 1	526/1912 (27.51%)	611/1578 (38.72%)	550/1371 (40.12%)	552/1328 (41.57%)	<.001^b^
	Month 3	427/1175 (36.34%)	467/1156 (40.40%)	428/1132 (37.81%)	475/1103 (43.06%)	.02
	Month 6	327/827 (39.54%)	395/893 (44.23%)	342/845 (40.47%)	414/943 (43.90%)	.12
	Month 12	306/730 (41.92%)	355/833 (42.62%)	314/845 (37.16%)	386/884 (43.67%)	.06
**Missing = smoking**						
	Month 1	526/4118 (12.77%)	611/4097 (14.91%)	550/4110 (13.38%)	552/4105 (13.45%)	.03
	Month 3	427/4118 (10.37%)	467/4097 (11.40%)	428/4110 (10.41%)	475/4105 (11.57%)	.20
	Month 6	327/4118 (7.94%)	395/4097 (9.64%)	342/4110 (8.32%)	414/4105 (10.09%)	.002^b^
	Month 12	306/4118 (7.43%)	355/4097 (8.66%)	314/4110 (7.64%)	386/4105 (9.40%)	.005^b^

^a^ Conditions were as follows: 1: a noninteractive smoking cessation guide, cigarette counter, and an online journal; 2: condition 1, plus individually timed email messages; 3: condition 2, plus an 8-session cognitive–behavioral mood management course; and condition 4: condition 3, plus a virtual participant-driven support group.

^b^ Significant, controlling for demographic characteristics (gender, age, education, race), language of the intervention (English or Spanish), level of addiction (Fagerström Test for Nicotine Dependence [FTND] score), and depression (Center for Epidemiologic Studies Depression scale [CES-D] score and presence of current or past major depressive episodes).

### Putting the Outcomes of a Fully Automated Trial in Perspective

**Figure 2:**
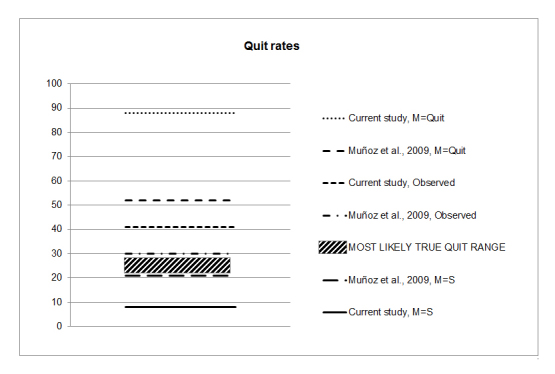
Most likely quit rate range extrapolated from the current trial and an identical trial with live follow-up (Muñoz et al [14]). M=Quit: missing observations are presumed quit ([reported quit + missing]/all assigned to condition); M=S: missing observations are presumed smoking (reported quit/all assigned to condition); Observed: missing observations are excluded (reported quit/[reported quit + reported smoking).

For the current trial, the quit rate at 12 months was 8%, assuming M=S. However, the *true* quit rate is unknown: 8% is clearly an underestimate, because the M=S is only an assumption and is highly conservative. As a thought experiment, if the opposite assumption is made—that all those with missing data have quit (M=Quit)—then the quit rate would be 88% (8% observed quit + 80% missing). The M=S is an underestimate of the true quit rate, but M=Quit is clearly an overestimate. Though we can say with confidence that the true quit rate resides between 8% and 88%, this is not very informative (Figure 2).

We can approximate the true quit rate by using the rates from the earlier [14] trial. Because the 2009 trial used exactly the same intervention (plus live follow-up), similar quit rates can be expected. To determine whether the two cohorts are similar, we used binary logistic regression with the same covariates as in the previous models to compare the two most similar subgroups in the two trials: those in the 2009 study who completed all four follow-ups after only an automated email reminder (and were thus never exposed to live follow-up), and those in the current study who also completed all four follow-ups. For these subgroups, the 12-month quit rates were not significantly different (46/96, 48% in the 2009 study vs 714/1326, 54% in the present study; Wald χ^2^_1_ = 1.6, *P* = .21, odds ratio = 0.75, 95% CI 0.48–1.17), which provides additional credence for the ability to extrapolate results of the current study in the context of the 2009 trial. Only two covariates crossed the significant threshold. One was depression history (Wald χ^2^_2_ = 13.8, *P =* .001), with participants without a history of depression appearing to quit at higher rates (603/1043, 57.8%) than those with past (92/213, 43.2%) or current (62/156, 40%) depression. The other was FTND score, with those scoring higher being less likely to quit (Wald χ^2^_1_ = 7.0, *P* = .01, odds ratio = 0.94, 95% CI 0.90–0.99).

The 2009 [14] study obtained an average quit rate of 21% at 12 months assuming M=S, or, assuming M=Quit, 52%. The true rate for the current study therefore most likely resides between 21% and 52%, which is considerably more informative than the 8%–88% interval (Figure 2).

 The interval can be narrowed down further. The observed quit rate for the current study at 12 months is 41%; in the 2009 [14], it was 30%. However, that 30% was based on both automated and live follow-up responders, and the reported quit rates of automated responders were about 70% higher than that of live responders (16.3% vs 9.8%, respectively, across all follow-ups). The current study’s observed quit rate (41%) is therefore an overestimate of the true quit rate, as everyone who provided data did so with automated follow-up. The most likely conclusion about the true quit rate in the current study is that the upper bound is 30% (observed quit rate in the 2009 study) and the lower bound is 21%, as illustrated in Figure 2.

## Discussion

In this paper we have highlighted the problems of attrition in international Internet trials, especially in the context of the M=S convention, and offered a way to reconcile the demands of needing to employ costly means of follow-up with the advantages that the breadth of a very-large-scale automated trial allows. By referencing the identically conducted trial, with the only difference being live follow-up for those who did not respond to automated email reminders, we estimated the true quit rate for the current trial to lie between 21% and 30% of participants. We also found that more complex versions of the intervention resulted in better cessation rates than a static online smoking cessation guide, suggesting that some level of complexity and personalization may be helpful in Internet interventions.

 Internet interventions are a relatively new form of health-promoting behavior change interventions that are likely to grow considerably due to the benefits of reach and cost effectiveness. To ensure that these interventions are improving health outcomes, they must be tested to ensure a strong evidence base. Indeed, Internet-based interventions are increasingly evidence based [40,41]. These interventions will reach a larger proportion of the world with increased Internet penetration [42] and improving technology such as integration of mobile devices and linkages with electronic health records. However, novel interventions call for novel methods of their evaluation, especially where conventional methods fall short.

One of the most significant benefits of Internet interventions is their cost effectiveness due to sustainability and nonconsumability. The cost of creating an effective Internet site for smoking cessation is relatively modest, about US $55,000 in our case. Conducting the randomized trials to evaluate its effectiveness costs much more. Once the original efficacy trial with live follow-up was completed, however, leaving the site open to conduct the fully automated randomized trial reported here was relatively inexpensive. We estimate that it cost about US $120 per participant who reported quitting successfully by at least one follow-up point. If the nicotine patch had been used, assuming a 20% quit rate with the patch, at a cost of US $3.91 per day [4] for 10 weeks, the cost per participant who quit successfully using the patch would have been about US $1370.

 The benefit of Internet intervention trials may be undervalued if methods for their evaluations underestimate their effects. Though sophisticated statistical procedures that may tackle missing data exist (eg, multiple imputation), they may not be accurate when the proportion of missing data is very large, as is often the case in fully automated Internet studies. Therefore, we have proposed a hybrid solution that includes conducting a smaller trial with aggressive follow-up using live methods (eg, phone calls) to assess for an intervention’s efficacy followed by a larger naturalistic trial to assess effectiveness. This method would allow for the rigorous testing of efficacy by ensuring high follow-up rates with a smaller trial. The larger trial would then allow one to take advantage of the wide reach and automated nature of Internet interventions to assess for the overall impact of the study by extrapolating results from the smaller trial onto a larger sample. Thus, the 2009 [14] study first established the efficacy of the intervention, and this paper has outlined the second step of our proposed method by highlighting effectiveness of the same intervention delivered in an automated format. These methods can also be applied to prevention trials that have similar issues in that they use dichotomous outcomes and similar conventions when people drop out of a study or are lost to follow-up. Applying a method that first assesses efficacy and then focuses on broad reach would better inform the potential impact of large-scale public health campaigns that are difficult to interpret due to difficulties with and cost of follow-up assessments.

There are limitations to our study and the way we have used our proposed 2-step method. In both studies, smoking was assessed via self-report rather than biomedical validation measures; however, this is the recommended approach in large-scale community trials [24]. Participants in the efficacy trial [14] were the first 500 Spanish speakers and 500 English speakers randomly assigned to the 4 study conditions. History effects were therefore not controlled, though the two cohorts were found to be comparable. In future studies, the follow-up cohort should be selected randomly across time from the large sample of participants in automated self-help studies. Lastly, the actual outcomes of participants who did not provide follow-up data are unknown. We have made the case that informed estimates can be made, when they are based on efficacy data in a subsample with rigorous follow-up. In some parts of the world, Internet access is available only to those of higher socioeconomic status. This is rapidly changing, however, with the growth of Internet penetration being the highest in the developing countries [42]. Finally, the majority of participants were non-US Spanish speakers. The results may not generalize to other populations.

 Internet-based interventions for health problems are becoming increasingly popular due to their enormous reach and cost effectiveness: no other medium permits conducting a randomized trial of an empirically supported intervention for over 16,000 individuals across 165 countries at such low cost. However, in testing these interventions via randomized controlled trials, particularly when assessing dichotomous outcomes, it is necessary to develop new methods of analysis that are able to fully reflect the true impact and effectiveness of large-scale, international public health Internet interventions.

Future directions involve carrying out outcome studies that are more generalizable to how Internet interventions would be used outside of a strict randomized trial context. Specifically, users of such sites are likely to pick and choose among intervention elements provided by the sites. Thus, the next step after randomized trials ought to be participant preference trials, in which users are provided access to all elements of the interventions that were found to be reasonably effective within a randomization context, and allowed to use the elements they prefer. Our team is conducting such a study, which we believe would best estimate the effectiveness of a self-help automated Internet intervention that would be made available at no charge to anyone in the world who wanted to use it.

Researchers in the Internet intervention field should consider adopting this approach, namely a progression of studies, from strict efficacy randomized trials (with live follow-ups to reduce attrition), to fully automated randomized trials (to approximate how a self-help site would be used), proceeding to participant preference effectiveness studies (in which all elements tested in the earlier randomized trials are made available to all participants). Such an approach would contribute to the use of evidence-based Internet interventions to reduce health disparities worldwide [43].

## Abbreviations

CES-D: Center for Epidemiologic Studies Depression scale
CI: confidence interval
FTND: Fagerström Test for Nicotine Dependence
LOCF: last observation carried forward
M=Quit: missing observations presumed quit
M=S: missing observations presumed smoking
MDE: major depressive episode

## Multimedia Appendix 1

CONSORT-EHEALTH V1.6 checklist [44].

## References

[1] World Health Organization (2008). WHO Report on the Global Tobacco Epidemic 2008: The MPOWER Package.

[2] Fiore MC, Smith SS, Jorenby DE, Baker TB (1994). The effectiveness of the nicotine patch for smoking cessation. A meta-analysis. JAMA.

[3] Lando HA, McGovern PG, Barrios FX, Etringer BD (1990). Comparative evaluation of American Cancer Society and American Lung Association smoking cessation clinics. Am J Public Health.

[4] Schroeder SA (2005). What to do with a patient who smokes. JAMA.

[5] Glasgow RE (2002). Evaluation of theory-based interventions: the RE-AIM model. Glanz K, Rimer BK, Lewis FM, editors. Health Behavior and Health Education: Theory, Research, and Practice. 3rd edition.

[6] Glasgow RE, Klesges LM, Dzewaltowski DA, Bull SS, Estabrooks P (2004). The future of health behavior change research: what is needed to improve translation of research into health promotion practice?. Ann Behav Med.

[7] Glasgow RE, Klesges LM, Dzewaltowski DA, Estabrooks PA, Vogt TM (2006). Evaluating the impact of health promotion programs: using the RE-AIM framework to form summary measures for decision making involving complex issues. Health Educ Res.

[8] Glasgow RE, Lichtenstein E, Marcus AC (2003). Why don't we see more translation of health promotion research to practice? Rethinking the efficacy-to-effectiveness transition. Am J Public Health.

[9] Glasgow RE, Nelson CC, Strycker LA, King DK (2006). Using RE-AIM metrics to evaluate diabetes self-management support interventions. Am J Prev Med.

[10] Glasgow RE, Orleans CT, Wagner EH (2001). Does the chronic care model serve also as a template for improving prevention?. Milbank Q.

[11] Glasgow RE, Vogt TM, Boles SM (1999). Evaluating the public health impact of health promotion interventions: the RE-AIM framework. Am J Public Health.

[12] Brendryen H, Kraft P (2008). Happy ending: a randomized controlled trial of a digital multi-media smoking cessation intervention. Addiction.

[13] Etter JF (2005). Comparing the efficacy of two Internet-based, computer-tailored smoking cessation programs: a randomized trial. J Med Internet Res.

[14] Muñoz RF, Barrera AZ, Delucchi K, Penilla C, Torres LD, Pérez-Stable EJ (2009). International Spanish/English Internet smoking cessation trial yields 20% abstinence rates at 1 year. Nicotine Tob Res.

[15] Muñoz RF, Lenert LL, Delucchi K, Stoddard J, Perez JE, Penilla C, Pérez-Stable EJ (2006). Toward evidence-based Internet interventions: A Spanish/English Web site for international smoking cessation trials. Nicotine Tob Res.

[16] Pike KJ, Rabius V, McAlister A, Geiger A (2007). American Cancer Society's QuitLink: randomized trial of Internet assistance. Nicotine Tob Res.

[17] Strecher VJ, McClure J, Alexander G, Chakraborty B, Nair V, Konkel J, Greene S, Couper M, Carlier C, Wiese C, Little R, Pomerleau C, Pomerleau O (2008). The role of engagement in a tailored web-based smoking cessation program: randomized controlled trial. J Med Internet Res.

[18] Strecher VJ, Shiffman S, West R (2006). Moderators and mediators of a web-based computer-tailored smoking cessation program among nicotine patch users. Nicotine Tob Res.

[19] Swartz LH, Noell JW, Schroeder SW, Ary DV (2006). A randomised control study of a fully automated internet based smoking cessation programme. Tob Control.

[20] Eysenbach G (2005). The law of attrition. J Med Internet Res.

[21] Saul JE, Schillo BA, Evered S, Luxenberg MG, Kavanaugh A, Cobb N, An LC (2007). Impact of a statewide Internet-based tobacco cessation intervention. J Med Internet Res.

[22] Velicer WF, Prochaska JO, Rossi JS, Snow MG (1992). Assessing outcome in smoking cessation studies. Psychol Bull.

[23] Strecher VJ, Becker MH, Clark NM, Prasada-Rao P (1989). Using patients' descriptions of alcohol consumption, diet, medication compliance, and cigarette smoking: the validity of self-reports in research and practice. J Gen Intern Med.

[24] Hughes JR, Keely JP, Niaura RS, Ossip-Klein DJ, Richmond RL, Swan GE (2003). Measures of abstinence in clinical trials: issues and recommendations. Nicotine Tob Res.

[25] Muñoz RF, Marín BV, Posner SF, Pérez-Stable EJ (1997). Mood management mail intervention increases abstinence rates for Spanish-speaking Latino smokers. Am J Community Psychol.

[26] National Cancer Institute (2002). Guia Para Dejar de Fumar. NIH Publication No. 02-3001.

[27] Pérez-Stable EJ, Sabogal F, Marín G, Marín BV, Otero-Sabogal R (1991). Evaluation of “Guia para Dejar de Fumar,” a self-help guide in Spanish to quit smoking. Public Health Rep.

[28] Lenert L, Muñoz RF, Perez JE, Bansod A (2004). Automated e-mail messaging as a tool for improving quit rates in an internet smoking cessation intervention. J Am Med Inform Assoc.

[29] Lewinsohn PM, Muñoz RF, Youngren MA, Zeiss AM (1992). Control Your Depression.

[30] Heatherton TF, Kozlowski LT, Frecker RC, Fagerström KO (1991). The Fagerström Test for Nicotine Dependence: a revision of the Fagerström Tolerance Questionnaire. Br J Addict.

[31] Muñoz RF (2010). University of California, San Francisco, School of Medicine, Latino Mental Health Research Program.

[32] Muñoz RF, McQuaid JR, González GM, Dimas J, Rosales VA (1999). Depression screening in a women's clinic: using automated Spanish- and English-language voice recognition. J Consult Clin Psychol.

[33] Spitzer RL, Williams JB, Kroenke K, Linzer M, deGruy FV, Hahn SR, Brody D, Johnson JG (1994). Utility of a new procedure for diagnosing mental disorders in primary care. The PRIME-MD 1000 study. JAMA.

[34] Vázquez FL, Muñoz RF, Blanco V, López M (2008). Validation of Muñoz's Mood Screener in a nonclinical Spanish population. Eur J Psychol Assess.

[35] Radloff LS (1977). The CES-D scale: a self-report depression scale for research in the general population. Appl Psychol Meas.

[36] Hall SM, Humfleet GL, Reus VI, Muñoz RF, Hartz DT, Maude-Griffin R (2002). Psychological intervention and antidepressant treatment in smoking cessation. Arch Gen Psychiatry.

[37] Hall SM, Muñoz RF, Reus VI (1994). Cognitive-behavioral intervention increases abstinence rates for depressive-history smokers. J Consult Clin Psychol.

[38] Hall SM, Muñoz RF, Reus VI, Sees KL, Duncan C, Humfleet GL, Hartz DT (1996). Mood management and nicotine gum in smoking treatment: a therapeutic contact and placebo-controlled study. J Consult Clin Psychol.

[39] Hall SM, Reus VI, Muñoz RF, Sees KL, Humfleet G, Hartz DT, Frederick S, Triffleman E (1998). Nortriptyline and cognitive-behavioral therapy in the treatment of cigarette smoking. Arch Gen Psychiatry.

[40] Marks IM, Cavanagh K, Gega L (2007). Hands-on Help: Computer-Aided Psychotherapy.

[41] Moser DJ, Reese RL, Hey CT, Schultz SK, Arndt S, Beglinger LJ, Duff KM, Andreasen NC (2006). Using a brief intervention to improve decisional capacity in schizophrenia research. Schizophr Bull.

[42] (2010). Miniwatts Marketing Group.

[43] Muñoz RF (2010). Using evidence-based internet interventions to reduce health disparities worldwide. J Med Internet Res.

[44] Eysenbach G, CONSORT-EHEALTH Group (2011). CONSORT-EHEALTH: Improving and Standardizing Evaluation Reports of Web-based and Mobile Health Interventions. J Med Internet Res.

