# Supplementary material for: Interpreting the Outcomes of Automated Internet-Based Randomized Trials: Example of an International Smoking Cessation Study
Source: J Med Internet Res. 2012 Feb 7;14(1):e5. doi: 10.2196/jmir.1829 (PMC3374542; doi:10.2196/jmir.1829)
Supplement: Supplementary file 1 [file jmir_v14i1e5_app1.pdf]

Manuscript Number 1829

Date completed 8/25/2011 14:50:29

by Ricardo Muñoz

Interpreting the outcomes of automated Internet-based randomized trials:
Example of an international smoking cessation study

TITLE

1a-i) Identify the mode of delivery in the title

"Internet-based"

1a-ii) Non-web-based components or important co-interventions in title

1a-iii) Primary condition or target group in the title

"smoking cessation study"

ABSTRACT

1b-i) Key features/functionalities/components of the intervention and comparator in the METHODS section of the ABSTRACT

"Objectives: To illustrate a method to interpret outcomes of large-scale fully automated worldwide Internet intervention trials.
Methods: A fully automated international Internet-based smoking cessation randomized controlled trial (RCT) was conducted in Spanish and English, with 16,430 smokers from 165 countries. The RCT replicated a published efficacy trial in which, to reduce follow-up attrition, 1,000 smokers were followed up by phone if they did not provide online follow-up data."

1b-ii) Level of human involvement in the METHODS section of the ABSTRACT

1b-iii) Open vs. closed, web-based (self-assessment) vs. face-to-face assessments in the METHODS section of the ABSTRACT

1b-iv) RESULTS section in abstract must contain use data

1b-v) CONCLUSIONS/DISCUSSION in abstract for negative trials

INTRODUCTION

2a-i) Problem and the type of system/solution

"A key benefit of Internet interventions is the automation of delivery. However, with few exceptions,[13, 18] most trials rely on live personnel to conduct follow-up assessments via phone or email contacts with participants. Insofar as interaction with live personnel may affect outcomes, these trials depart from the “fully automated” framework. The two main reasons for sacrificing the benefits of automation and fidelity of administration, while incurring considerable costs to conduct live follow-up, are attrition and the analytical convention of imputing smoking status to missing data when presenting cessation trial outcomes."
"With live follow-up (e.g., phone calls), it is possible for geographically limited Internet trials to obtain follow-up rates of up to 78%, which is comparable to face-to-face trials.[21] However, live follow-ups for large-scale worldwide Internet trials are costly and logistically difficult. Conversely, allowing the logistical limitations of live follow-up procedures to constrain the number of participants compromises scope and reach, limiting the public health applications of an Internet trial.
We propose one possible model of structuring an Internet trial that may help assess effectiveness of a trial once efficacy is established. In 2009, Muñoz and colleagues reported on the outcome of a web-based smoking cessation trial conducted in Spanish and English (n=1,000).[14] Live follow-up was used with those who failed to provide data after an automated reminder, obtaining follow-up rates of 68% at 12 month follow-up. At 12 months, 20% of Spanish- and 21% of English-speaking participants were no longer smoking (M=S). After 1,000 participants were randomized, live follow-up ended but the rest of the online intervention study was left exactly the same, with the goal of conducting a larger trial to demonstrate the demand for and the reach of the intervention as delivered in a fully automated format. Here, we report on the results of the fully automated portion of that trial.
Conducting a smaller and logistically feasible live follow-up trial followed by or concurrently with a larger fully-automated trial can address the concerns of cost vs. scope mentioned previously. The goal of the current study is therefore to illustrate the use of this approach in interpreting the outcomes of a fully automated trial. We use the outcomes of the Muñoz et al.[14] trial to interpret the results of the current fully automated trial, and we test three hypotheses to determine whether a more complex intervention increases quit rates."

2a-ii) Scientific background, rationale: What is known about the (type of) system

"Attrition
The motivation needed to enter traditional face-to-face trials is high: people either actively seek them out, respond to an advertisement by calling and visiting a clinic, or are directly recruited from pre-existing registries based on demographic, behavioral or clinical factors. In contrast, those signing up for a web-based trial generally do so via a web search and clicking on a link. Of the thousands who visit the Website, few will elect to join, fewer still will make adequate use of the intervention, and only a minority will respond to automated follow-up invitations. The difference in effort involved to enter an Internet trial versus a face-to-face trial makes comparisons between the two problematic. Website visitors are more akin to persons reading an advertisement for a trial, most of whom will not actually call or visit the study clinic. Those filling out an online eligibility questionnaire are similar to those calling a phone number to inquire more about a traditional outcome study. Signing up for an online trial takes little effort; though many online participants are likely curious about the Internet trial, they may not be as committed to participating as those signing up after traveling to a study clinic. Once entry into the study takes place, it is extremely easy to drop out of an Internet trial, since there has been no direct personal contact with study staff. The field needs to reconsider how best to interpret findings which involve large attrition to systematically study the effectiveness of Internet interventions as they would be routinely used in practice, rather than as part of a well-staffed randomized controlled trial.
Attrition is a recognized concern in Internet trials,[20] which affects interpretation of results in two ways: 1) if most participants drop out, the remaining sample is highly self-selected, and may not be representative of the original visitors, and 2) if participants fail to complete the intervention, but respond to follow-up, the outcome data won’t represent the intervention’s potential. Of course, there are also parallels in face-to-face trials: participants are also highly self-selected and not a representative sample of all who suffer from the disorder being treated.

The “missing=smoking” convention
The usual strategy for determining quit rates in a cessation trial is the “missing=smoking” (M=S) convention, a variant of the intent-to-treat (ITT) analyses, which presumes that all participants unreachable at follow-up are smoking. This is similar to the last observation carried forward (LOCF) convention, however, the M=S convention is more conservative, for two reasons. First, the LOCF convention permits “responded-to-treatment” observations to be carried forward as well as “nonresponse”, whereas M=S presumes that every dropout is a treatment failure. Second, because cessation trial outcomes are dichotomous (quit vs. did not quit), degree of response/nonresponse (e.g., fewer cigarettes) cannot be captured by M=S. The outcomes of cessation trials therefore are largely dependent on the completeness of follow-up data. For example, suppose the “true” quit rate for a hypothetical intervention is 20%. Three trials assessing the effect of that intervention with follow-up rates of 100%, 70%, and 40% would yield M=S outcomes of 20%, 14%, and 8%, respectively, prompting widely differing conclusions about efficacy. Since 14-22% can be expected with a nicotine patch [4] and 4-8% can be obtained with a placebo patch, the possible M=S outcome implications vary significantly. Because automated Internet trials have inherently high dropout rates, the M=S convention may be more reflective of follow-up success than of treatment efficacy."

METHODS

3a) CONSORT

"The goal of the current study is therefore to illustrate the use of this approach in interpreting the outcomes of a fully automated trial. We use the outcomes of the Muñoz et al.[14] trial to interpret the results of the current fully automated trial, and we test three hypotheses to determine whether a more complex intervention increases quit rates."
"Hypotheses
Three specific hypotheses regarding the outcome of the intervention were retained from the 2009 [14] study and tested in the fully automated sample:
(1) Conditions 2, 3, and 4 will outperform Condition 1;
(2) Condition 4 will obtain the best quit rates, followed by Condition 3, followed by Condition 2, followed by Condition 1; and
(3) Conditions 3 and 4 (containing mood management) will outperform Conditions 1 and 2."

3b-i) Bug fixes, Downtimes, Content Changes

4a-i) Computer / Internet literacy

4a-ii) Open vs. closed, web-based vs. face-to-face assessments:
"Recruitment procedures were the same as described elsewhere.[14] Google AdWords ads were the main source of recruiting participants."
"Briefly, visitors to the site completed brief demographics and eligibility questionnaires. Consenting participants completed baseline questionnaires. To select out one-time visitors, participants were asked to return 3 times over the next 7 days and report the number of cigarettes smoked."
4a-iii) Information giving during recruitment

4b-i) Report if outcomes were (self-)assessed through online questionnaires
"Participants were sent automated follow-up assessment emails at 1, 3, 6 and 12 months after their quit date. Only self-report smoking data were gathered"
4b-ii) Report how institutional affiliations are displayed

5-i) Mention names, credential, affiliations of the developers, sponsors, and owners

5-ii) Describe the history/development process

5-iii) Revisions and updating

5-iv) Quality assurance methods

5-v) Ensure replicability by publishing the source code, and/or providing screenshots/screen-capture video, and/or providing flowcharts of the algorithms used

5-vi) Digital preservation

5-vii) Access
Participants accessed the website at their convenience. The following phrases address the other elements of this section.
"Google AdWords ads were the main source of recruiting participants."
"Participants were not paid for their participation in the study."
5-viii) Mode of delivery, features/functionalities/components of the intervention and comparator, and the theoretical framework
As in the 2009 trial, [14] participants were randomized into four conditions. Condition 1 contained the most basic elements, and Conditions 2-4 incrementally added further elements.
Participants "were automatically randomized into 4 conditions, and were given access to the website. "
"As in the 2009 trial, [14] participants were randomized into four conditions. Condition 1 contained the most basic elements, and Conditions 2-4 incrementally added further elements.
1.A non-interactive, static smoking cessation guide (Guía para dejar de fumar [24-26]), a cigarette counter, and an online journal.
2.Condition 1, plus Individually Timed Email Messages (ITEMs) -- pre-programmed emails with links to sections of smoking cessation guide timed to quit date.[28]
3.Condition 2, plus an 8-session cognitive-behavioral mood management course (based on Lewinsohn et al.[29]).
4.Condition 3, plus a virtual participant-driven unmoderated support group."
5-ix) Describe use parameters

5-x) Clarify the level of human involvement

5-xi) Report any prompts/reminders used
"Participants were sent automated follow-up assessment emails at 1, 3, 6 and 12 months after their quit date."
"in the present study live follow-up was not used."
"Individually Timed Email Messages (ITEMs) -- pre-programmed emails with links to sections of smoking cessation guide timed to quit date.[28]"
5-xii) Describe any co-interventions (incl. training/support)
N/A
6a-i) Online questionnaires: describe if they were validated for online use and apply CHERRIES items to describe how the questionnaires were designed/deployed

6a-ii) Describe whether and how "use" (including intensity of use/dosage) was defined/measured/monitored

6a-iii) Describe whether, how, and when qualitative feedback from participants was obtained

7a-i) Describe whether and how expected attrition was taken into account when calculating the sample size

7b) CONSORT
N/A
8a) CONSORT
"The website used a pre-programmed algorithm to randomize participants using stratified randomization, with gender and history of MDEs as stratification variables."
8b) CONSORT
"The website used a pre-programmed algorithm to randomize participants using stratified randomization, with gender and history of MDEs as stratification variables."
9) CONSORT
"The website used a pre-programmed algorithm to randomize participants using stratified randomization, with gender and history of MDEs as stratification variables."
10) CONSORT
"The website used a pre-programmed algorithm to randomize participants using stratified randomization, with gender and history of MDEs as stratification variables."
11a-i) Specify who was blinded, and who wasn't
"Eligible participants viewed and e-signed a consent document, which detailed the study procedures, including randomization."
11a-ii) Discuss e.g., whether participants knew which intervention was the "intervention of interest" and which one was the "comparator"

11b) CONSORT
In this trial, each condition added an element, as described in the following paragraph:
"Condition 1 contained the most basic elements, and Conditions 2-4 incrementally added further elements.
1.A non-interactive, static smoking cessation guide (Guía para dejar de fumar [25-27]), a cigarette counter, and an online journal.
2.Condition 1, plus Individually Timed Email Messages (ITEMs) -- pre-programmed emails with links to sections of smoking cessation guide timed to quit date.[28]
3.Condition 2, plus an 8-session cognitive-behavioral mood management course (based on Lewinsohn et al.[29]).
4.Condition 3, plus a virtual participant-driven unmoderated support group (an asynchronous bulletin board)."
12a) CONSORT

"To test Hypotheses 1 (Condition 1 will result in worst outcomes) and 3 (conditions with mood management will result in better outcomes), repeated binary logistic regressions were conducted. The quit rates were predicted from the intervention condition assignment (1 versus others for Hypothesis 1; 1 and 2 versus 3 and 4 for Hypothesis 3), covarying participant demographic characteristics (gender, age, education, race), language (English or Spanish), depression (CES-D score and presence of current or past MDE), and level of addiction (FTND). These analyses were conducted twice: once with the Missing=Smoking assumption, and the other – with observed data (without the M=S assumption).
To test Hypothesis 2 -- that intervention conditions yielded incrementally better outcomes, binary logistic regression models were constructed, predicting the 7 day quit rate at 1, 3, 6, and 12 months. The model predictors were the same as used for repeated measures analyses, described above. As above, these analyses were conducted twice: once with the Missing=Smoking assumption, and the other – with observed data (without the M=S assumption).
Due to the considerable size of the sample (n = 16,430), we elected to report significance only if a P-value less than 0.01 is obtained, to reduce Type I error."

12a-i) Imputation techniques to deal with attrition / missing values

The goal of the paper is to interpret online randomized trials that are likely to have high degree of attrition. The method we are describing in this paper involves a close comparison with an identical trial with live follow-up (and therefore lower attrition).

12b) CONSORT

N/A

RESULTS

13a) CONSORT

This information can be found in the CONSORT diagram for progression of participants through the fully automated Internet Stop Smoking Trial (Figure 1).

13b) CONSORT

"The progression of participants through the study is outlined in Figure 1. Of the over 150,000 participants who visited our website, 78,623 provided enough data to evaluate their eligibility, 28,703 signed consent, and 16,430 completed baseline assessments and the washout period and were randomized.
The current study relied solely on automated emailed reminders to obtain follow-up data. For month 1 follow-up, 40.0% of participants provided data. This number was reduced to 30.4%, 23.2%, and 22.0% for follow-ups at months 3, 6, and 12, respectively. These numbers were comparable to those obtained in the earlier [14] study, where 38%, 30%, 27%, and 23% of participants who never received any live follow-up, returned at months 1, 3, 6, and 12, respectively."
The reasons for attrition are not known.

13b-i) Attrition diagram

14a) CONSORT

"Participants were recruited from November 2005 to September 2009. "
"Participants were sent automated follow-up assessment emails at 1, 3, 6 and 12 months after their quit date."

14a-i) Indicate if critical "secular events" fell into the study period

14b) CONSORT

The trial was stopped in order to transition to a participant preference trial, which is described in another paper, currently under review at JMIR (Muñoz, Aguilera, Schueller, Leykin, & Pérez-Stable. From Online Randomized Controlled Trials to Participant Preference Studies: Morphing the San Francisco Stop Smoking Site into a Worldwide Smoking Cessation Resource).

15) CONSORT

"Table 1. Participant characteristics, by condition."

15-i) Report demographics associated with digital divide issues

Some this information is reported in Table 1.

16-i) Report multiple "denominators" and provide definitions

We discuss the issue of denominators in the Introduction:
"The "missing=smoking" convention
The usual strategy for determining quit rates in a cessation trial is the "missing=smoking" (M=S) convention, a variant of the intent-to-treat (ITT) analyses, which presumes that all participants unreachable at follow-up are smoking. This is similar to the last observation carried forward (LOCF) convention, however, the M=S convention is more conservative, for two reasons. First, the LOCF convention permits "responded-to-treatment" observations to be carried forward as well as "nonresponse", whereas M=S presumes that every dropout is a treatment failure. Second, because cessation trial outcomes are dichotomous (quit vs. did not quit), degree of response/nonresponse (e.g., fewer cigarettes) cannot be captured by M=S. The outcomes of cessation trials therefore are largely dependent on the completeness of follow-up data. For example, suppose the "true" quit rate for a hypothetical intervention is 20%. Three trials assessing the effect of that intervention with follow-up rates of 100%, 70%, and 40% would yield M=S outcomes of 20%, 14%, and 8%, respectively, prompting widely differing conclusions about efficacy. Since 14-22% can be expected with a nicotine patch and 4-8% can be obtained with a placebo patch, the possible M=S outcome implications vary significantly. Because automated Internet trials have inherently high dropout rates, the M=S convention is more reflective of follow-up success than it is of treatment efficacy."
We present both the intent-to-treat (missing=smoking) and observed (available data) analyses in Table 2.

16-ii) Primary analysis should be intent-to-treat

17a) CONSORT

"Several differences between treatment conditions were noted. With observed data (i.e. without M=S assumption), at 1-month follow-up, Condition 1 performed significantly poorer than all other conditions, in partial support for Hypothesis 1 (Wald Chi-Square(3)=80.7, P<.001). No significant differences between conditions were noted at months 3 (Wald Chi-Square(3)=10.3, P=.02), 6 (Wald Chi-Square(3)=5.8, P=.12), and 12 (Wald Chi-Square(3)=7.49, P=.06). With M=S analyses, significant differences were observed at months 6 (Wald Chi-Square(3)=14.8, P=.002) and 12 (Wald Chi-Square(3)=13.0, P=.005), such that conditions 2 and 4 outperformed conditions 1 and 3.
Observing the quit rates, it is clear that Hypothesis 2 – that conditions will result in incremental improvements in quit rates – is not supported. To test the two other hypotheses, repeated measures logistic regressions were conducted, with the same covariates as simple logistic regressions above. Hypothesis 1 was largely supported. With observed data, condition 1 resulted in lower quit rates than conditions 2, 3, and 4 (Wald Chi-Square(1)=30.1, P<.001, B=-.28, 95%CI: -.39 – -.18). A similar result was observed with M=S data, though the result failed to cross the significance level set for this study (Wald Chi-Square(1)=6.1, P=.01, B=-.12, 95%CI: -.21 – -.02). Hypothesis 3 – that mood management conditions will result in higher quit rates -- was supported only with observed data (Wald Chi-Square(1)=9.5, P=.002, B=-.14, 95%CI: -.23 – -.05), but not with the M=S data (Wald Chi-Square(1) =.00, P=.96, B=.00, 95%CI: -.08 – 0.78)."
For binary logistic regression, only group differences (all 4 conditions) are reported. Group differences do not yield effect sizes (odds rations).

17a-i) Presentation of process outcomes such as metrics of use and intensity of use

17b) CONSORT

"Several differences between treatment conditions were noted. With observed data (i.e. without M=S assumption), at 1-month follow-up, Condition 1 performed significantly poorer than all other conditions, in partial support for Hypothesis 1 (Wald Chi-Square(3)=80.7, P<.001). No significant differences between conditions were noted at months 3 (Wald Chi-Square(3)=10.3, P=.02), 6 (Wald Chi-Square(3)=5.8, P=.12), and 12 (Wald Chi-Square(3)=7.49, P=.06). With M=S analyses, significant differences were observed at months 6 (Wald Chi-Square(3)=14.8, P=.002) and 12 (Wald Chi-Square(3)=13.0, P=.005), such that conditions 2 and 4 outperformed conditions 1 and 3.
Observing the quit rates, it is clear that Hypothesis 2 – that conditions will result in incremental improvements in quit rates – is not supported. To test the two other hypotheses, repeated measures logistic regressions were conducted, with the same covariates as simple logistic regressions above. Hypothesis 1 was largely supported. With observed data, condition 1 resulted in lower quit rates than conditions 2, 3, and 4 (Wald Chi-Square(1)=30.1, P<.001, B=-.28, 95%CI: -.39 – -.18). A similar result was observed with M=S data, though the result failed to cross the significance level set for this study (Wald Chi-Square(1)=6.1, P=.01, B=-.12, 95%CI: -.21 – -.02). Hypothesis 3 – that mood management conditions will result in higher quit rates -- was supported only with observed data (Wald Chi-Square(1)=9.5, P=.002, B=-.14, 95%CI: -.23 – -.05), but not with the M=S data (Wald Chi-Square(1) =.00, P=.96, B=.00, 95%CI: -.08 – 0.78)."
For binary logistic regression, only group differences (all 4 conditions) are reported. Group differences do not yield effect sizes (odds rations).
We do not report absolute effect sizes.

18) CONSORT

Only pre-specified analyses were performed.

18-i) Subgroup analysis of comparing only users

19) CONSORT

None noted

19-i) Include privacy breaches, technical problems

19-ii) Include qualitative feedback from participants or observations from staff/researchers

DISCUSSION

20-i) Typical limitations in ehealth trials

"There are limitations to our study and the way we have utilized our proposed two-step method. In both studies, smoking was assessed via self-report rather than biomedical validation measures; however this is the recommended approach in large-scale community trials.[24] Participants in the “efficacy” trial[14] were the first 500 Spanish speakers and 500 English speakers randomized to the four study conditions. History effects were therefore not controlled, though the two cohorts were found to be comparable. In future studies, the follow-up cohort should be selected randomly across time from the large sample of participants in automated self-help studies. Lastly, the actual outcomes of participants who did not provide follow-up data are unknown. We have made the case that informed estimates can be made, when they are based on efficacy data in a subsample with rigorous follow-up. In some parts of the world, Internet access is only available to those of higher SES. This is rapidly changing, however, with the growth of Internet penetration being the highest in the developing countries.[42] Finally, the majority of participants were non-US Spanish speakers. The results may not generalize to other populations."

21-i) Generalizability to other populations

21-ii) Discuss if there were elements in the RCT that would be different in a routine application setting

22-i) Restate study questions and summarize the answers suggested by the data, starting with primary outcomes and process outcomes (use)

" In this paper we have highlighted the problems of attrition in international Internet trials, especially in the context of the M=S convention, and offered a way to reconcile the demands of needing to employ costly means of follow-up with the advantages that the breadth of a very large scale automated trial allows. By referencing the identically conducted trial, with the only difference being live follow-up for those who did not respond to automated email reminders, the true quit rate for the current trial was estimated to lie between 21% to 30% of participants. We also found that more complex versions of the intervention resulted in better cessation rates than a static online smoking cessation guide, suggesting that some level of complexity and personalization may be helpful in Internet interventions. "

22-ii) Highlight unanswered new questions, suggest future research

Other information

23) CONSORT

ClinicalTrials.gov NCT00721786

24) CONSORT

A more thorough description of the trial can be found in the following publication:
Muñoz RF, Barrera AZ, Kevin Delucchi, Penilla C, Pérez-Stable EJ. International Spanish/English Internet Smoking Cessation Trial Yields 20% Abstinence Rates at One Year. Nicotine and Tobacco Research. 2009;11:102534 PMID: 19640833.
For the full trial protocol, please contact the corresponding author (RFM).

25) CONSORT

"This research was supported by grant 13RT-0050 from the Tobacco-Related Disease Research Program, by an infrastructure grant from the University of California Committee on Latino Research to the University of California/San Francisco General Hospital Latino Mental Health Research Program (LMHRP; Muñoz, P.I.), by grants from the Tobacco Research Network Program, Fogarty International Center, National Institute of Drug Abuse (No. TW05935, Pérez-Stable, P.I.) and from the National Cancer Institute for Redes en Acción (U01CA86117, Pérez-Stable, P.I), National Institutes of Health, by NIMH Training grant to Adrian Aguilera (MH 5T32MH018261-27; Patricia Arean, P.I.), and by NIMH grant 5K08MH091501 (Leykin, P.I.). The authors thank the Center for Health and Community (Nancy Adler, Director) for providing office space and additional resources. Special thanks go to Google, Inc. for awarding the UCSF/SFGH LMHRP an AdWords grant (Muñoz, P.I.), which provided us with the ability to recruit smokers worldwide using Google sponsored links. "

X26-i) Comment on ethics committee approval

x26-ii) Outline informed consent procedures

X26-iii) Safety and security procedures

X27-i) State the relation of the study team towards the system being evaluated
